# Efficient oil–water separation coating with robust superhydrophobicity and high transparency

**DOI:** 10.1038/s41598-022-06220-9

**Published:** 2022-02-09

**Authors:** Baiyi Chen, Rongrong Zhang, Hexuan Fu, Jiadai Xu, Yuan Jing, Guohe Xu, Bin Wang, Xu Hou

**Affiliations:** 1 State Key Laboratory of Physical Chemistry of Solid Surfaces, College of Chemistry and Chemical Engineering, Xiamen University, Xiamen, 361005 China; 2 College of Science and Technology, Hebei Agricultural University, Cangzhou, 061100 China; 3 Key Laboratory of Advanced Technologies of Materials (Ministry of Education), School of Materials Science and Engineering, Southwest Jiaotong University, Chengdu, 610031 China

**Keywords:** Environmental chemistry, Materials chemistry, Environmental sciences, Materials science

## Abstract

There has been a growing interest in oil–water separation due to the massive economic and energy loss caused by world-wide oil spill. In the past decades, a new type of superhydrophobic surface has been developed for the efficient oil–water separation, but its large-scale use is significantly limited by its expensive, sophisticated, and fragile roughness structure. Meanwhile, to handle complex operating conditions, the transparency of the superhydrophobic surface has been more attractive due to its potential visual oil–water separation and optical application scenarios. Herein, we showed a simple and versatile strategy to fabricate superhydrophobic coating with robustness and high transparency. Subsequently, this multifunctional superhydrophobic coating was utilized for oil–water separation and indicated excellent separation efficiency. In this strategy, candle soot composed of carbon nanoparticles was deposited onto the substrate and used as a rough surface template. Then, a filmy and hard silica shell was modified onto this template via chemical vapor deposition to reinforce the roughness structure. Following, this soot-silica coated substrate was calcined in air to remove the candle soot template. Finally, based on a rational surface design, this robust silica coating achieved excellent superhydrophobicity thereby showing inherently oil–water separation benefits. This reinforced superhydrophobic coating presented robust superhydrophobicity even after 410 s sand impacting with the height of 40 cm and 20 cycles of sandpaper abrasion. Also, it retained excellent oil–water separation efficiency even after reuses.

## Introduction

Superhydrophobicity refers to the phenomenon with the contact angle between water and the corresponding surface greater than 150° and the rolling angle less than 10°^1,2^. From ancient cave paintings to modern microfluidic devices^3^, numerous researches and practical applications have been devoted to deepening the understanding of the superhydrophobic surface. The inherently merits of superhydrophobic surface, such as its self-cleaning, drag reduction, condensation-heat transfer, anti-fouling and anti-icing^4–9^, have shown great potentials for broad applications in industry, biomedicine, military, and many other fields^10–14^. However, limited on the precise micro-nano hierarchical structure which normally relies on the sophisticated micro- and nano-particles physical accumulation^15–18^, the improvement of the superhydrophobic surface remains challenging, especially its functional integration and durability in practical application scenarios.

To handle these problems, many efforts have been devoted to developing robust and multi-functional superhydrophobic surfaces to adapt to the complex and harsh environments^19–21^. Recently, Seeger et al.^22^ has created a hierarchical architecture combining micro- and nano-scale structure to protect the superhydrophobic surface with precise roughness from the mechanical damage. Glass beads (75 μm) were partially embedded in the low-density polyethylene matrix, followed by coating with nanoscale silicone filaments. Furtherly, TiO_2_ nanoparticles were synthesized in situ on the silicone nanofilaments as photocatalyst. Consequently, it has been demonstrated this hierarchical composite surface possessed self-cleaning property and enhanced mechanical durability. Additionally, Deng et al.^23^ developed a robust superhydrophobic surface by structuring it with a hierarchical micro-nano system. The interconnected micro-structured surface frame acted as an ‘armour’ to provide the robustness, and the fluorinated nano-structured silica clusters housed in the micro-structured frame ensured its water repellency. Especially, this strategy can be applied to various substrates and showing strong superhydrophobicity even after abrasion by sandpaper or sharp steel blade. However, the robust function-integrated superhydrophobic coating is still in its early stage, and there are many challenges to be done for its development, such as the complex and cumbersome preparation^24^, high-cost^25^ and poor functional stability^26^, which have severely limited its large-scale application. Thus, the exploitation of a durable and function-integrated superhydrophobic coating with simple and versatile preparation is essential to make up for the lack of the conventional superhydrophobic coating.

In this work, we have shown a simple and versatile strategy to develop a new type of robust superhydrophobic coating with high transparency. Firstly, candle soot was physically accumulated onto the quartz wafer surface forming a rough template. Then, a silicon dioxide layer was deposited on the template by chemical vapor deposition of SiCl_4_ to create a hard and rough coating. Based on a rational surface design, this robust coating achieved excellent superhydrophobicity thereby showing inherently oil–water separation benefits and broke the limitations of conventional superhydrophobic surface. This robust superhydrophobic coating fabricated in this way not only greatly improved its abrasion resistance, but also had low cost and simple operation, and could be mass-produced and popularized. Moreover, this highly transparent superhydrophobic coating possessed excellent optical transmittance to facilitate the real-time observation of the separation process in the oil–water separation device and make target adjustments. It is believed that this transparent superhydrophobic coating has potential applications in various aspects such as isolation film, protective glasses, lens coating and touch screen.

## Materials and methods

### Materials

Candle (Kameyama Co., Ltd., Japan), Oil blue 35 (Askul Co., Ltd., Japan), P1200 silicon carbide waterproof abrasive paper (Eagle Brand, Kovax Co., Ltd., Japan) with the particle size of ~ 15.3 μm, stainless steel mesh with the average pore size of 800 μm (Huirui wire mesh Co., Ltd, China), Scarlet 4GE (Askul Co., Ltd., Japan), gasoline (Komeri Co., Ltd., Japan) and sea sands. The laboratory solvents and Trichloro (1H, 1H, 2H, 2H-perfluorooctyl) silane were purchased from Nacalai tesque.

### Fabrication of the transparent, robust and superhydrophobic coating

To achieve a robust, transparent and superhydrophobic coating, the substrate (quartz wafer or stainless-steel mesh) was first thoroughly washed with ethanol and deionized water thoroughly, and dried in an oven at 60 °C. The cleaned substrate was then placed over the candle flame until a few microns thick layer of soot was deposited. Subsequently, the soot coated substrate together with SiCl_4_ were placed in a drier for chemical vapor deposition about 2 h. As a result, like the Stöber reaction^27^, a porous silica shell was formed on the soot coated substrate by hydrolysis and condensation of SiCl_4_. Then, through calcination at 600 °C for half an hour in air, the candle soot composed of carbon nanoparticles thermally degraded and diffused through the silica shell gradually. Consequently, a superhydrophilic and hollow silica shell was produced on the substrate. To modify the surface into superhydrophobic, the silica shell was performed chemical vapor deposition in a drier together with about 1 mL of trichloro (1H, 1H, 2H, 2H-perfluorooctyl) silane for 2 h at 60 °C. Finally, a superhydrophobic coating was obtained on substrate caused by stable oxane bonds formed between abundant hydroxide radical on silica shell and silane.

Furthermore, in order to investigate the oil–water separation application of this transparent, robust and superhydrophobic coating, a rounded stainless-steel mesh was utilized as substrate to perform the modification. In particular, both sides of the mesh were modified to ensure its superhydrophobicity entirely.

### Robustness tests

Falling sand abrasion and sandpaper abrasion tests were used to study the mechanical stability of the superhydrophobic coating. In the falling sand abrasion test, sea sands with the average particle size of 300–500 μm were employed to impact the multifunctional superhydrophobic coating with a height of 40 cm, while the substrate was held at 30° to the horizontal surface. After a certain time of impact, this coating was observed and its water contact angle was measured. In the sandpaper abrasion test, a modified quartz wafer under a weight of 100 g was placed face-down to a P1200 silicon carbide sandpaper and moved cyclically to test the mechanical stability in dynamic application scenarios.

### Oil–water separation

To investigate the oil–water separation application of the multifunctional superhydrophobic coating, the oil–water mixture was formed by mixing dichloromethane (5 mL) dyed with Solvent Blue 35 and water (5 mL) dyed with Scarlet 4GE. The oil–water separation device was prepared by placing a round modified stainless steel mesh between the split type funnel, and then placed it on the top of a flask. During the oil–water separation, the dichloromethane-water mixture was used as a representative and quickly poured into the separation device, the oil phase was allowed to permeate through the modified stainless-steel mesh while the water phase was blocked. Finally, the separation efficiency was investigated to quantitatively evaluate separation property of our device, which was defined as the water content in oil phase after separation and measured by Karl Fischer moisture titrator.

### Characterization

Surface morphologies of samples were carried out on a Hitachi s-4800 (Hitachi, Japan) Field-emission Scanning Electron Microscope (SEM) at 15.0 kV. Moisture in oil phase was analyzed by Karl Fischer Moisture Titrator (MKC-60 Kyotoelectronics) Manufacturing Co. LTD., Japan). The contact angle (CA) was characterized on an OCA100 instrument (Dataphysics, Germany) in air. The Fourier transform infrared (FT-IR) analysis was carried out on in-situ Fourier transform infrared spectrometer (Bruker Vertex 70 V, Germany) using KBr pellets in the range of 400–4000 cm^−1^. Water droplets around 4 μL in volume were placed on multiple areas on the surface of samples. Photographic images were taken with a Nikon camera (D 5000).

## Results and discussion

**Figure 1:**
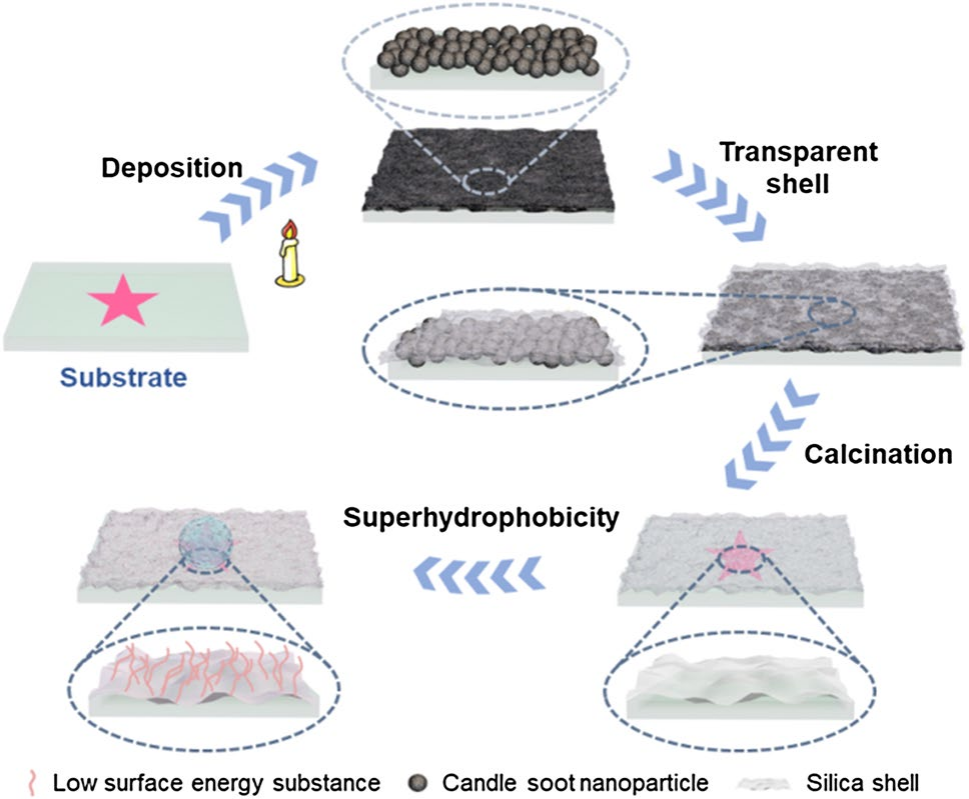
Schematic illustration of preparation and properties of the transparent and robust superhydrophobic coating.

According to the illustration shown in Fig. 1, the cheap and accessible candle soot were utilized as a rough surface template to directly deposit onto the substrate. The candle soot was composed of carbon nanoparticles forming by incompletely burned paraffin, which loosely accumulated on the substrate and formed a rough surface. Following, a hard and porous silica shell was deposited on the candle soot template through chemical vapor deposition of SiCl_4_ like Stöber reaction, which completely replicate the roughness of the template and formed a robust rough surface. Because the thickness of the silica shell was much smaller than the wavelength of visible light (420–700 nm), it showed good transparency. After removal of the carbon nanoparticles template by calcination in air, the hard and transparent silica shell was reserved on substrate. Due to the abundant hydroxyl on this silica shell, it can be easily modified into superhydrophobicity through grafting trichloro (1H, 1H, 2H, 2H-perfluorooctyl) silane. As a result, a transparent silica coating with robust superhydrophobicity was obtained by rational surface design.

### Surface modification and characterization

**Figure 2:**
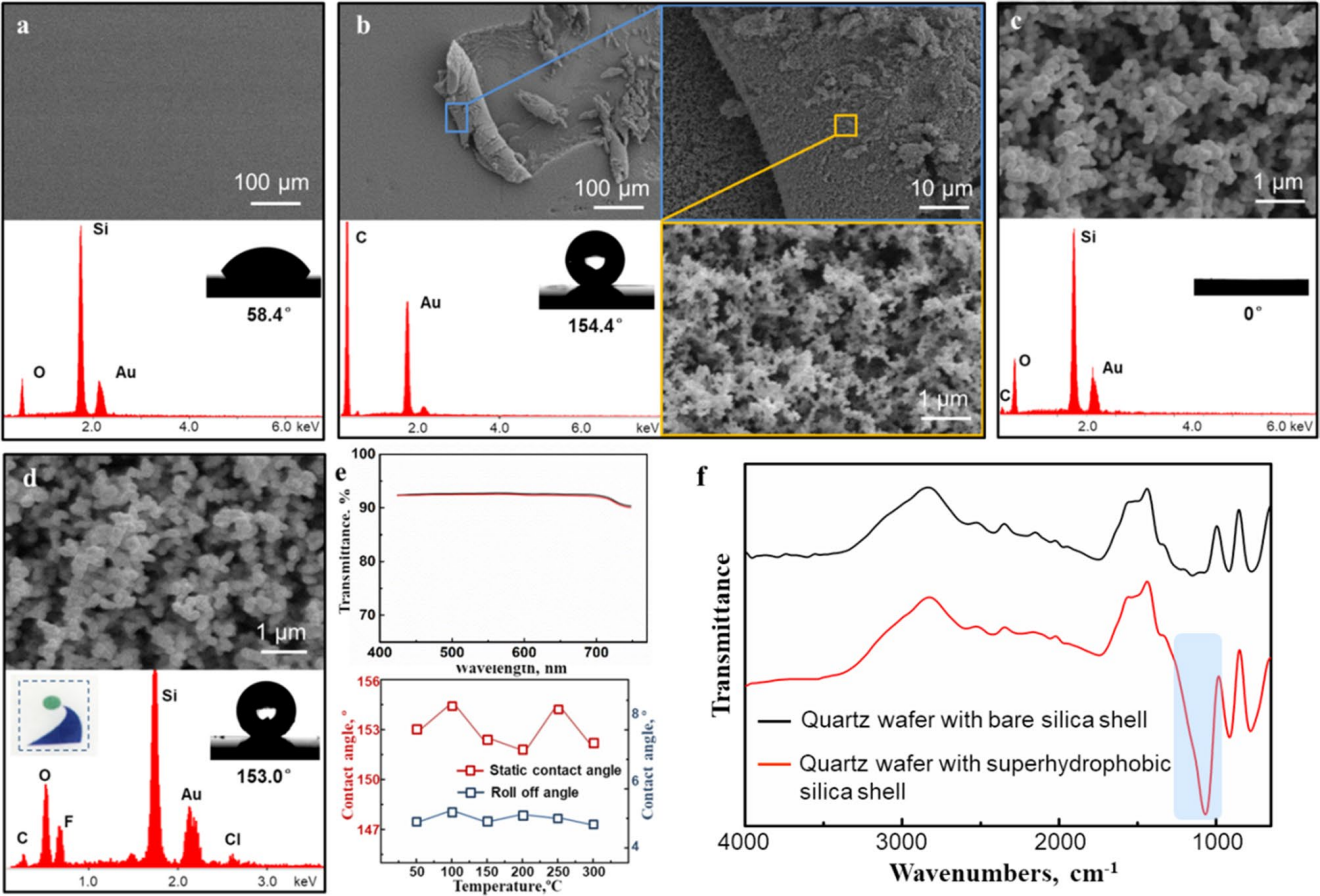
The SEM images, CA, EDS and FT-IR analysis of different quartz wafer samples: (**a**) the bare quartz wafer, (**b**) the quartz wafer with the candle soot rough template, (**c**) the quartz wafer with the silica shell reinforced rough template, (**d**) the quartz wafer with the hollow and superhydrophobic silica shell, (**e**) the visible light transmittance and thermal stability of the superhydrophobic coating, (**f**) FT-IR analysis of the quartz wafers with bare and modified silica shell.

During preparation of this transparent and robust superhydrophobic coating, the surface wettability has changed significantly. Taking the quartz wafer sample as a representative, its surface modifications have been successfully demonstrated (Fig. 2). As shown in Fig. 2a, the bare quartz wafer was washed carefully with dry ethanol, ethanol-deionized water (v/v 1:1) and deionized water in turn, and then characterized by SEM, CA and energy dispersive spectrum (EDS). The SEM images indicated that the surface morphology of bare quartz wafer was homogeneous and smooth, and the EDS analysis only detected the signals of Si and O, the main elements that constituted quartz wafer, indicating a thoroughly clean surface. Meanwhile, the bare quartz wafer showed a hydrophilic surface with the water droplet contact angle of 58.4°, which was mainly due to the presence of hydroxyl on its surface.

Following, to prepare the candle soot template, the abluent quartz wafer was held above the flame of a candle for 3–5 s until a black soot layer with a few micrometers thickness was deposited. As can be seen in Fig. 2b, the SEM and EDS analysis indicated that the candle soot had formed a loose and porous layer, which was consisted entirely of carbon nanoparticles with the diameter of 30 to 40 nm. Meanwhile, a rough surface formed by accumulating candle soot nanoparticles can be observed on the quartz wafer. As is well recognized, the surface chemical composition and morphology are considered to be the key factors for obtaining superhydrophobicity on solid surface^28^. The surface chemical composition with low surface energy ensures its hydrophobicity, and the rough surface morphology with micro-nano hierarchical structure amplifies hydrophobicity into superhydrophobicity furtherly^29^. For instance, the surface of an abluent plastic sheet is only naturally hydrophobic, but not superhydrophobic (CA above 150°). However, it is interesting to note that a rough surface accumulated by hydrophobic nanoparticles can be amplified into superhydrophobicity. Therefore, the rough surface formed by accumulating candle soot nanoparticles showed a significant superhydrophobicity with water contact angle of 154.4°. Due to the weak physical interaction between the soot nanoparticles, this soot deposition layer was fragile. As water droplets rolled down from it, the soot nanoparticles were easily taken off by the droplets until nearly all of the deposits were removed, causing a wettability transition of the surface. Inspired by the promising morphology and super-wettability of candle soot deposits, a novel method to replicate and enhance the roughness structure was adopted^30^. In this method, a silica shell was prepared to cover the candle soot layer by chemical vapor deposition of SiCl4, as shown in Fig. 1. As can be seen from Fig. 2c obviously, the average particle diameter of the candle soot increased slightly after the chemical vapor deposition, but its original morphology has been completely replicated. This has demonstrated that the roughness structure of the candle soot layer was not destroyed by chemical vapor deposition of SiCl4. Thus, the silica shell achieved superhydrophilicity (CA about 0°) due to the hydrophilicity of the silica and the roughness structure of candle soot layer. The SEM image and EDS analysis of the silica shell after calcination in air was shown in Fig. 2d. As can be seen from this figure, the candle soot template composed by carbon nanoparticles has been removed during calcination, but its rough and porous surface morphology was completely reserved by the hard silica shell. After surface modification by grafting with trichloro (1H, 1H, 2H, 2H-perfluorooctyl) silane, the silica shell surface showed a static contact angle of 153.0°. Because of the extremely low adhesion interaction between water and this superhydrohobic surface, the water droplets would be difficult to deposit and immediately roll off. In some special application scenarios, such as goggles or touch screens, the superhydrophobic coating would require transparency, heat stability and mechanical robustness. By calcination, the candle soot carbon nanoparticles disappeared and cavities were formed. Since the thickness of the hollow silica shell was smaller than the wavelength of visible light, this shell was highly transparent (shown as the inset). The visible light transmittance of the quartz wafers before and after modification with transparent superhydrophobic coating were measured and shown in Fig. 2e top, the superhydrophobic coating modified quartz wafer showed almost the same transparence with the bare quartz wafer. Meanwhile, this transparent superhydrophobic coating showed excellent thermal stability, which remained superhydrophobicity even after 1 h calcination under high temperature up to 300ºC (Fig. 2e bottom). Additionally, in FT-IR analysis, the quartz wafer with modified silica shell showed the stretching vibration peak of C–F bonds at ~ 1100 cm-1 compared with the bare silica shell, which has demonstrated the successful modification of trichloro (1H, 1H, 2H, 2H-perfluorooctyl) silane on the silica shell (Fig. 2f).

### Robustness

**Figure 3:**
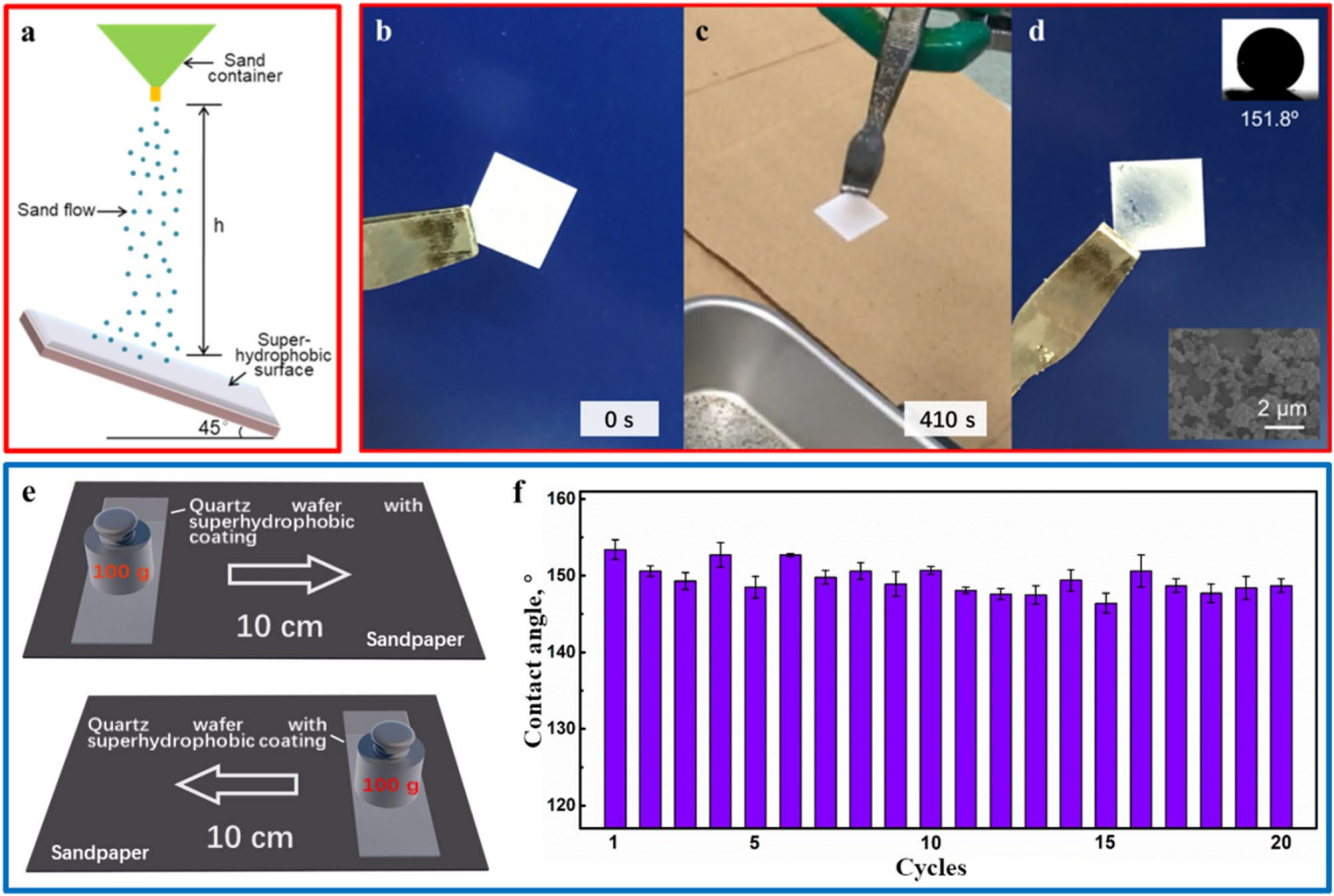
The falling sand abrasion and sandpaper abrasion tests of the superhydrophobic coating: (**a**) schematic diagram of the falling sand abrasion test; the superhydrophobic coating before (**b**), during (**c**) and after (**d**) the falling sand abrasion test, and its CA and SEM images were shown in the insets, (**e**) schematic diagram of the sandpaper abrasion test, (**f**) the mechanical stability of the superhydrophbic coating under sandpaper abrasion test.

When exposed to the natural environment, the superhydrophobic coating should be endowed mechanical robustness against the harsh application scenarios. Therefore, the falling sand abrasion and sandpaper abrasion tests were performed on this superhydrophobic coating to characterize its mechanical stability. Sand particles with an average diameter of 300 to 500 um were used to impact the superhydrophobic coating directly from a height of 40 cm (Fig. 3a), and the corresponding impact energy was 1.1 × 10^−4^ J/particle. As shown in Fig. 3b and c, the superhydrophobic silica shell were strong enough to fully withstand the impact of sand particles in 410 s. As the impact time increases, holes were eventually formed at the impacted area, and the silica shell was partially destroyed (Fig. 3d). In spite of this, the SEM image of the partially destroyed superhydrophobic coating indicated that the microscopic morphology of the cavities had hardly changed (Fig. 3d). Due to the self-similarity of the silica shell, this coating remained superhydrophobicity (CA = 151.8°) until the silica shell was completely removed from the substrate after continuous impact. In order to study the mechanical stability of this superhydrophobic coating in dynamic application scenarios, a modified quartz wafer was used for the sandpaper abrasion test. As shown in Fig. 3e, the wafer with superhydrophobic coating was placed face-down to the sandpaper under a weight of 100 g and moved back and forth for 10 cm, which was defined as one abrasion cycle. Meanwhile, water contact angle of the superhydrophobic coating after each abrasion cycle were shown in Fig. 3f. As can be observed, even after 20 cycles of sandpaper abrasion, this coating still remained superhydrophobicity, which verified its robustness once again. The mechanical stability of the superhydrophobic coating mainly depended on the thickness of the silica shell, which would increase with the thickness, but at the expense of the coating’s transparency.

### Oil–water separation

**Figure 4:**
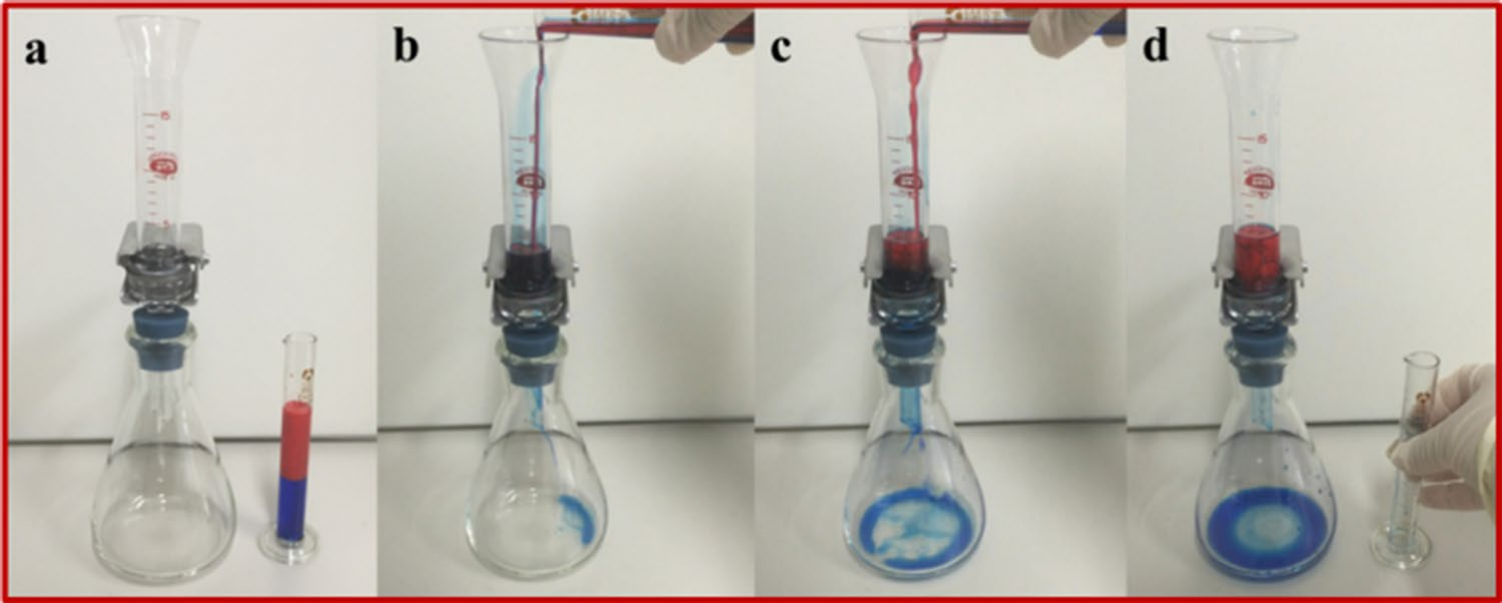
The oil–water separation application of the superhydrophobic coating: (**a**) color-dyed oil–water mixture was prepared to be separated by the separation device. (**b**) The oil–water mixture was mixed uniformly and poured into the device. (**c** and **d**) The oil–water mixture was successfully separated.

**Figure 5:**
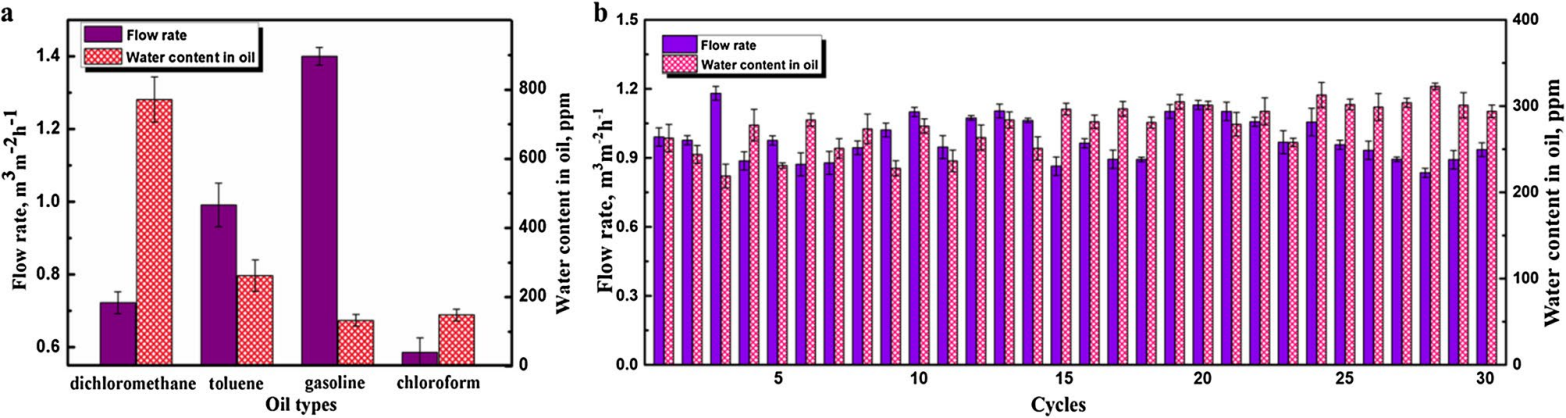
Oil–water separation application. (**a**) Separation efficiencies and flow rates with different oil–water mixtures. (**b**) Cycle stability of the separation device.

This superhydrophobic coating can be also used for oil–water separation due to its durable superhydrophobicity under oil. Thus, we utilized the strategy mentioned above to prepare an oil–water separation device with the stainless-steel mesh as substrate. Dichloromethane-water mixture was used as the representative to study the oil–water separation efficiency and oil flow rate, the results were shown in Figs. 4 and 5.

Both sides of the stainless-steel mesh were coated by the supehydrophobic silica shell as the oil–water separation mesh, and then the as-prepared mesh was imbedded into a split type filter. The filter was finally placed on a flask as shown in Fig. 4a. Following, the same volume (5 mL) of blue-dyed oil and red-dyed water was uniformly mixed and poured into the filter rapidly. Driven by gravity only, blue-dyed oil penetrated through the mesh immediately and red-dyed water was prevented (Fig. 4b, c). The oil–water mixture was completely separated; this was because the durable superhydrophobicity of the silica shell coated mesh even immersed under oil. The hydrophobic-oleophilic shell held a stable oil layer on its roughness surface, which constructed a 3-phase interface with water and prevented water from penetrating through the separation mesh (Fig. 4d). In addition, after oil–water separation course, the superhydrophobicity of the modified mesh was tested again. Water droplets did not wet or even contaminate the surface, demonstrating the reusability of the superhydrophilic surface.

According to the Cassie and Baxter theory^31^, the three-phase interface is necessary for achieving superhydrophobicity. During the oil–water separation, the water–oil-solid system also constructed a three-phase interface, which only allowed the oil through but repelled water off. Ultimately, the oil–water mixture was successfully separated only dependent on gravity by utilizing the feature of this superhydrophobic coating, indicating the low energy consumption and easy operation of this strategy.

Due to the frequent offshore oil spills, it is very necessary to spread the oil–water separation application to different oil–water mixtures^32,33^. We chose chloroform, toluene, gasoline and dichloromethane as the oil phases to mix with water. The separation efficiency was investigated to quantitatively evaluate separation property of our device, which was defined as the water content in oil phase after separation and measured by Karl Fischer moisture titrator. There were large differences of the separation efficiencies between different oil–water mixtures, this was mainly because water showed different solubility in different oil phases. The water contents in oil phases after separation were tested and shown in Fig. 5a, the separated gasoline, toluene, chloroform and dichloromethane presented water contents of 133 ppm, 262 ppm, 148 ppm and 77 ppm, respectively. Additionally, we also calculated the flow rates of different oil phases during separation through measuring its passing time, the results have been also shown in Fig. 5a. As can be observed, the toluene-water and gasoline-water mixtures showed the peak flow rates of 0.991 and 1.4 m^3^ m^−2^ h^−1^, which indicated the oil kinds influenced the penetrating rate significantly. For chloroform, its mixture needed longer time for stratification after mixing thoroughly. Therefore, it showed a relatively low flow rate of 0.586 m^3^ m^−2^ h^−1^. After that, the gasoline-water mixture was used as a representative and continuously separated for 30 times to investigate the cycle stability of this separation device, and the separation efficiencies and flow rates were further studied (Fig. 5b). For each separation cycle, the water content of the separated gasoline was below 320 ppm. In addition, the flow rate stabilized at about 1.05 m^3^ m^−2^ h^−1^, which verified the availability of this separation device. To sum up, the results indicated that this superhydrophobic coating has exhibited excellent comprehensive performance, not only in robustness and transparency, but also in oil–water separation applications.

## Conclusion

In summary, we have designed a highly transparent, robust and superhydrophobic coating using a versatile and simple straightforward template method, and its oil–water separation application has been demonstrated. In this work, the cheap and accessible candle soot composed of carbon nanoparticles was utilized as a rough template to obtain the hard and transparent silica shell with roughness. Following, the candle soot rough template was removed through calcination in air, thus a hollow and transparent silica shell was obtained. Furtherly, this silica shell with abundant hydroxyl was modified into superhydrophobic by grafting low surface energy substance, and ultimately used for oil–water separation. At the same time, the transparency, robustness and oil–water separation properties of this superhydrophobic coating were investigated. The results showed this transparent and robust superhydrophobic coating was successfully prepared and its water contact angle was 153.0°. The reinforced coating presented a mechanical robustness withstanding of 410 s sand impact. Additionally, the superhydrophobic coating revealed excellent separation efficiency and cycle stability in oil–water separation application. It remained good oil flow rate and oil–water separation efficiency even after 6 times reuses. It can be believed that this transparent and robust superhydrophobic coating could be potentially used in optical and visual application scenarios where suffer from harsh and oily environments, such as the goggles, building facade, visual oil–water separation device and touch screen.
